# Serum thyroxine and thyrotropin concentrations decrease with severity of nonthyroidal illness in cats and predict 30‐day survival outcome

**DOI:** 10.1111/jvim.15917

**Published:** 2020-10-01

**Authors:** Mark E. Peterson, Danielle L. Davignon, Nicole Shaw, Eric Dougherty, Mark Rishniw, John F. Randolph

**Affiliations:** ^1^ Animal Endocrine Clinic New York New York USA; ^2^ College of Veterinary Medicine Cornell University Ithaca New York USA; ^3^ Veterinary Emergency and Referral Group Brooklyn New York USA; ^4^ The Cat Practice New York New York USA; ^5^ Veterinary Information Network Davis California USA; ^6^ Present address: Veterinary Medical Center 5841 Ridge Street East Syracuse NY

**Keywords:** euthyroid sick syndrome, feline, hypothyroidism, thyroid‐stimulating hormone

## Abstract

**Background:**

In cats, nonthyroidal illness affects serum thyroid hormone concentrations. Serum thyroxine (T_4_) and triiodothyronine (T_3_) concentrations commonly decrease, whereas free T_4_ (fT_4_) concentrations vary unpredictably. Limited information exists regarding effects on serum thyrotropin (thyroid‐stimulating hormone [TSH]) concentrations in cats with nonthyroidal illness syndrome (NTIS).

**Objectives:**

To characterize alterations in thyroid function that develop in cats with NTIS and to correlate these alterations with severity and outcome of the nonthyroidal illness.

**Animals:**

Two hundred and twenty‐two cats with NTIS and 380 clinically normal cats of similar age and sex.

**Methods:**

Prospective, cross‐sectional study. All cats had serum T_4_, T_3_, free T_4_, and TSH concentrations measured. Cats were grouped based on illness severity and 30‐day survival.

**Results:**

Cats with NTIS had lower serum T_4_ and T_3_ concentrations than did normal cats (*P* < .001). Serum fT_4_ and TSH concentrations did not differ between groups. Serum T_4_, T_3_, and fT_4_ concentrations progressively decreased with increasing disease severity (*P* < .001). The 56 cats that died had lower T_4_, T_3_, and TSH concentrations than did the 166 survivors, with no difference in fT_4_ concentration. Multivariable logistic regression modeling indicated that serum T_4_ and TSH concentrations both predicted survival (*P <* .02).

**Conclusions and Clinical Importance:**

Cats with NTIS commonly develop low serum T_4_, T_3_, and TSH concentrations, the prevalence and extent of which increases with disease severity. Clinicians should consider evaluating thyroid function in cats with severe NTIS, because doing so could help determine probability of successful treatment responses before investing considerable time, effort, and finances in addressing the underlying disease.

**Abbreviations**
CI: confidence interval
fT_4_: free thyroxine
NTIS: nonthyroidal illness syndrome
ROC: receiver operating characteristic
T_3_: triiodothyronine
T_4_: thyroxine
TSH: thyroid‐stimulating hormone

## 1 INTRODUCTION

In humans, a variety of acute and chronic illnesses can alter the results of commonly used thyroid hormone function tests, such as serum total thyroxine (T_4_), free thyroxine (fT_4_), triiodothyronine (T_3_), and thyrotropin (thyroid‐stimulating hormone [TSH]) concentrations.^1^, ^2^, ^3^ This condition, known as the nonthyroidal illness syndrome (NTIS, previously termed “sick euthyroid syndrome”), is not a primary thyroid disorder but instead results from changes in secretion of TSH, as well as altered secretion, transport, metabolism, tissue uptake, and action of the thyroid hormones.^3^, ^4^, ^5^, ^6^ A likely adaptive response to the systemic illness, NTIS attempts to decrease peripheral tissue energy expenditure and minimize metabolic demands during the stress of the illness.^3^, ^4^, ^5^

Nonthyroidal illness can have marked effects on thyroid function tests. Human patients, especially those with severe or critical illness, commonly develop low serum T_4_ and T_3_ concentrations.^1^, ^2^, ^3^, ^7^, ^8^ Similarly, several nonthyroidal illnesses suppress serum T_4_ and T_3_ to low concentrations in dogs.^9^, ^10^, ^11^, ^12^ In both humans and dogs, serum fT_4_ concentrations, when measured by equilibrium dialysis, usually remain within the reference interval.^1^, ^2^, ^3^, ^8^ Most human patients with NTIS initially have normal serum TSH concentrations, but many will develop low TSH concentrations, especially those with severe illness.^1^, ^2^, ^3^, ^8^, ^13^ Approximately 10% to 15% of human patients will develop high serum TSH concentrations, particularly during the recovery phase of their illness.^1^, ^8^, ^14^, ^15^, ^16^ Similarly, dogs with NTIS usually maintain normal serum TSH concentrations, but occasionally have high serum TSH concentrations.^9^, ^10^, ^17^ In both human and dogs with NTIS, the finding of low serum T_4_ or fT_4_ concentrations, together with high TSH concentrations, complicates evaluation of thyroid function and increases the risk for misdiagnosis of primary hypothyroidism.^1^, ^2^, ^3^, ^11^, ^18^

In both humans^3^, ^19^, ^20^, ^21^, ^22^, ^23^ and dogs^10^, ^12^ with NTIS, development of low serum T_4_ and T_3_ concentrations increases the likelihood of death, a finding that might be useful as a prognostic indicator. Furthermore, in both humans and dogs, finding of low serum TSH concentrations has predicted mortality.^12^, ^16^, ^22^, ^24^, ^25^, ^26^

Few studies have examined the relationship between thyroid function and mortality in cats with NTIS.^27^, ^28^, ^29^, ^30^ In 2 studies that examined cats with a variety of nonthyroidal diseases,^27^, ^28^ cats that died or were euthanized had lower serum T_4_ concentrations than did cats that survived, suggesting that serum T_4_ concentrations may also be indicative of survival outcome. Similarly, a recent study of cats with panleukopenia reported that low serum T_4_ concentrations were associated with poor outcome.^30^ To our knowledge, no study has evaluated if serum T_3_ or TSH concentrations can help predict survival outcome in cats with NTIS.

As in humans and dogs, recent studies have demonstrated the utility of serum TSH concentrations for diagnosing cats with iatrogenic and naturally occurring hypothyroidism.^31^, ^32^, ^33^, ^34^ However, only limited data on serum TSH concentrations have been reported in a small number of cats with NTIS associated with chronic kidney disease, in which serum TSH concentration were within the reference interval.^35^, ^36^ If cats with NTIS do occasionally develop high serum TSH concentrations, this finding could lead to an erroneous diagnosis of hypothyroidism, as reported in humans and dogs with NTIS. ^1^, ^2^, ^3^, ^11^, ^18^

We sought to better determine the effect of nonthyroidal illness on commonly used serum pituitary‐thyroid function tests (T_4_, T_3_, fT_4_, TSH) in cats. Furthermore, we sought to determine the effect of severity of illness and disease category on serum thyroid hormone and TSH concentrations, as well as to examine whether abnormalities in any of these hormones could predict patient outcome and survival.

## 2 MATERIALS AND METHODS

### 2.1 Study design and selection of cats

We enrolled 2 groups of client‐owned cats for this prospective cross‐sectional study, which included cats with nonthyroidal illness and clinically normal cats. Cats with a history of hyperthyroidism were excluded. Ethical approval for the study was obtained from our institution's animal use and care committee, and blood collection was performed after informed owner consent.

#### 2.1.1 Clinically normal, euthyroid cats

We recruited 380 clinically normal cats as controls, as well as to establish institutional reference intervals for serum T_4_, T_3_, fT_4_, and TSH concentrations. These cats were considered healthy based on an unremarkable client history, physical examination (ie, none had palpable thyroid nodules or showed signs of hypothyroidism^34^), and routine laboratory testing (ie, CBC, serum biochemistry profile, and urinalysis).

#### 2.1.2 Cats with NTIS

Two hundred and twenty‐two cats were diagnosed with NTIS on the basis of results of history, physical examination, laboratory testing (eg, CBC, serum biochemistry profile, urinalysis, FeLV, and feline immunodeficiency virus status), and, variably, as required by the primary disease process, imaging (eg, radiography, ultrasonography, computerized tomography, or magnetic resonance imaging), and cytology or histologic examination. All cats were considered to be euthyroid on the basis of results of history, physical examination (ie, none had palpable thyroid nodules or showed signs of hypothyroidism^34^), and diagnostic tests that established a specific diagnosis of nonthyroidal disease. None of these cats had received medications within the 2‐week period before blood sampling that might affect serum thyroid hormone concentrations (eg, nonsteroidal anti‐inflammatory agents, sulfonamides, phenobarbital, tricyclic antidepressants, glucocorticoids), and none had received methimazole or thyroid hormone replacement.^37^, ^38^, ^39^, ^40^

Cats with NTIS were allocated to 3 groups based on disease severity (ie, mild, moderate, and severe). This judgment was made by the clinician who examined the cat, in consultation with the primary author (M.E. Peterson), and was based on a number of factors, including the cats' clinical signs, results of laboratory testing, duration of illness, need for hospitalization, response to treatment, and survival. In terms of hospitalization requirement and duration, we allocated cats to the mild disease group if the clinician believed that the cat could be treated as an outpatient. We allocated cats to the moderate disease group if the clinician recommended brief hospitalization (regardless of the owner's permission to hospitalize the cat). We allocated cats to the severe disease group if the clinician recommended intensive hospital care, whether or not the owner accepted these recommendations.

The 222 cats also were divided into 10 groups based on their primary category of disease (ie, cardiac, dermatologic, endocrine, gastrointestinal, hepatic, infectious, neoplastic, neurologic, respiratory, and urologic/renal disease). In cats that suffered from >1 disease, the selected category was based on the primary or most severe issue, as determined both by the clinician examining the cat and primary author (M.E. Peterson). Finally, these cats also were classified according to 30‐day survival outcome (ie, alive or dead within 30 days of serum thyroid hormone testing).

### 2.2 Assays for thyroid hormone and thyrotropin (TSH) concentrations

Serum concentrations of total T_4,_ total T_3_, fT_4_ by dialysis, and TSH were determined by assays validated for use in cats as previously described.^41^ The sensitivity (ie, limit of quantification) of the each assay was 6.5 nmol/L for T_4_, 0.55 nmol/L for T_3_, 5 pmol/L for fT_4_, and 0.03 ng/mL for TSH.^41^ For the T_3_ and TSH assays, analytic sensitivity was not low enough to distinguish low‐normal from low concentrations (ie, many clinically normal cats have undetectable serum T_3_ and TSH concentrations when measured by these assays).^41^

All blood samples for hormone assays were centrifuged within 1 hour after collection; serum was separated and stored at ≤4°C until assayed by a commercial laboratory (Antech Diagnostics, Lake Success, New York) the next day.

### 2.3 Data and statistical analyses

Data were assessed for normality using the D'Agostino‐Pearson test and by visual inspection of graphical plots.^42^ Data were not normally distributed; therefore, all analyses were performed using nonparametric tests.

Undetectable serum TSH concentration was defined as <0.03 ng/mL and all undetectable serum TSH concentrations were assigned an arbitrary value of 0.02 ng/mL for continuous data analysis, as previously described.^41^ Similarly, all undetectable serum T_4_ concentrations (<6.5 nmol/L) were assigned an arbitrary value of 3.5 nmol/L, whereas all undetectable serum T_3_ concentrations (<0.55 nmol/L) were assigned an arbitrary value of 0.45 nmol/L for continuous data analysis.

We used data from our 380 clinically normal cats to establish our institutional reference intervals for serum concentrations of T_4_, T_3_, fT_4_, and TSH using a nonparametric method to identify the central 95th percentile interval (ie, 2.5 through 97.5th percentile range).^43^, ^44^ Table 1 shows our reference intervals with 90% confidence intervals (CIs) for the thyroid hormones determined using this method.

**TABLE 1:** Reference intervals for total thyroxine (T_4_), total triiodothyronine (T_3_), free T4 by dialysis, and TSH established in 380 clinically normal cats

Hormone	Lower limit of RI (90% CI)	Upper limit of RI (90% CI)
Total T_4_ (nmol/L)	13 (11.6‐15.4)	49 (45‐51.5)
Total T_3_ (nmol/L)	0.45 (0.45‐0.45)	1.25 (1.09‐1.57)
Free T_4_ by dialysis (pmol/L)	11.5 (10‐13)	50 (46‐53)
TSH (ng/mL)	<0.03 (0.02‐0.02)	0.30 (0.25‐0.36)

Abbreviations: CI, confidence interval; RI, reference interval; TSH, thyroid‐stimulating hormone.

Results for continuous data (eg, serum thyroid hormone and TSH concentrations) are expressed as median (25th‐75th percentile) and represented graphically as boxplots (Tukey method).^45^ Results for qualitative data are expressed as ratio (breed, sex) or number (%) of cats. Continuous variables were compared between 2 groups by use of the Mann‐Whitney *U* test and for ≥3 groups by the Kruskal‐Wallis test, followed by the Dunn multiple comparisons test.^46^, ^47^ Categorical variables were compared among groups using the Chi‐square test.

To evaluate the predictive value of serum thyroid hormone and TSH concentrations on survival, we performed logistic regression using the 30‐day survival outcome as the dependent variable, and the serum T_4_, T_3_, fT4, and TSH concentrations as independent variables.^48^, ^49^ For this analysis, we entered serum T_4_ and fT4 concentrations as continuous variables and serum T_3_ and TSH concentrations as dichotomous (binary) variables (data coded 0 for undetectable concentrations; 1 for detectable concentrations). The significance of each explanatory variable was tested using the Wald test. Results of the model are reported in terms of adjusted odds ratios with 95% confidence intervals (95% CIs) for each explanatory variable. To evaluate the model's ability to discriminate between groups, we calculated the area under the receiver operating characteristic (ROC) curve. We also generated a classification table to compare the observed and predicted survival outcome and determine the percentage of cases correctly classified using the logistic regression model.^48^

For all analyses, statistical significance was defined as *P* ≤ .05. All statistical analyses were performed using proprietary statistical software (GraphPad Prism, version 7.0; GraphPad Software, La Jolla, California; MedCalc, version 19.2, MedCalc Statistical Software, Ltd, Ostend, Belgium).

## 3 RESULTS

### 3.1 Cat groups

#### 3.1.1 Clinically normal, euthyroid cats

These 380 cats ranged in age from 1 to 18 years (median = 10 years; 25th‐75th percentile = 8‐13 years). Breeds included domestic longhair and shorthair (328 cats; 86.3%), American Shorthair (9 cats), Siamese (8 cats), Persian (7 cats), Maine Coon (5 cats), Tonkinese (4 cats), Ragdoll (3 cats), Russian Blue (3 cats), Abyssinian (2 cats), and Balinese, Bengal, Burmese, Bombay, British shorthair, Chartreux, Egyptian Mau, Himalayan, Japanese Bobtail, Ocicat, and Scottish Fold (1 cat each). Of these cats, 195 (51%) were female and 185 were male; all had been neutered.

#### 3.1.2 Cats with NTIS

The 222 cats with NTIS ranged in age from 1 to 19 years (median = 11.0 years; 25th‐75th percentile = 7‐14 years). Breeds included domestic longhair and shorthair (192 cats; 86.5%), Maine Coon (6 cats), Siamese (5 cats), Persian (4 cats), Abyssinian (2 cats), American shorthair (2 cats), Russian Blue (2 cats), Himalayan (2 cats), Tonkinese (2 cats) and Balinese, British shorthair, Burmese, Ocicat, and Ragdoll (1 cat each). Of these, 118 (53%) were male and 104 were female; all had been neutered. The age, breed, and sex distribution of the 222 cats with NTIS did not differ from that of the 380 clinically normal cats.

The severity of illness was categorized as mild in 82 cats (37%), moderate in 72 (32%), and severe in 68 (31%). Of the 222 cats, 52 (23%) were diagnosed with urologic/renal disease, 45 (20%) with neoplastic disease, 28 (13%) with gastrointestinal disease, 22 (10%) with hepatic disease, 20 (9%) with endocrine disease other than hypothyroidism (primarily diabetes mellitus), 17 (8%) with infectious disease, 16 (7%) with cardiac disease, 10 (5%) with respiratory tract disease, 9 (4%) with neurologic disease, and 3 (1%) with dermatologic disease (Table 2).

**TABLE 2:** Serum thyroxine (T_4_), triiodothyronine (T_3_), free T_4_, and TSH in 222 cats with NTIS grouped into 10 categories of disease and severity of illness

NTIS group (no. of cats)	Serum T_4_ (nmol/L)	Serum T_3_ (nmol/L)	Serum fT_4_ (pmol/L)	Serum TSH (ng/mL)	Mild (no. of cats)	Moderate (no. of cats)	Severe (no. of cats)
Renal (52)	23.2 (14.2‐27.0)	0.46 (0.46‐0.61)	26 (16‐32)	0.07 (0.02‐0.10)	25	12	15
Neoplastic (45)	20.6 (14.2‐25.7)	0.46 (0.46‐0.46)	29 (21‐37)	0.06 (0.02‐0.14)	15	12	18
Gastrointestinal (28)	24.1 (19.3‐28)	0.46 (0.46‐0.52)	28 (23‐39)	0.05 (0.02‐0.08)	14	12	2
Hepatic (22)	16.1 (10.3‐25.4)	0.46 (0.46‐0.46)	30 (26‐47)	0.03 (0.02‐0.07)	4	8	10
Endocrine (20)	10.9 (7.7‐15.1)	0.50 (0.46‐0.73)	20 (15‐24)	0.05 (0.02‐0.08)	1	8	11
Infectious (17)	18.0 (10.3‐25.7)	0.46 (0.46‐0.46)	30.2 (20‐39)	0.05 (0.03‐0.10)	3	9	5
Cardiac (16)	30.2 (25.1‐31.9)	0.46 (0.46‐0.56)	30.7 (27‐33)	0.06 (0.04‐0.10)	10	2	4
Respiratory (10)	27.0 (19.0‐30.9)	0.46 (0.46‐0.66)	26 (19‐29)	0.03 (0.02‐0.10)	7	3	0
Neurologic (9)	18.0 (10.9‐25.7)	0.46 (0.46‐0.55)	37 (18‐39)	0.05 (0.03‐0.08)	1	6	2
Dermatologic (3)	27 (9‐27)	0.46 (0.46‐0.62)	26 (13‐45)	0.14 (0.02‐0.22)	2	0	1

*Note*: All results for T_4_, fT_4_, and TSH are listed as median (25th‐75th percentile).

Abbreviations: NTIS, nonthyroidal illness syndrome; TSH, thyroid‐stimulating hormone.

### 3.2 Serum thyroid hormone and TSH concentrations in cats with NTIS and clinically normal cats

#### 3.2.1 Serum T_4_ concentrations

**FIGURE 1:**
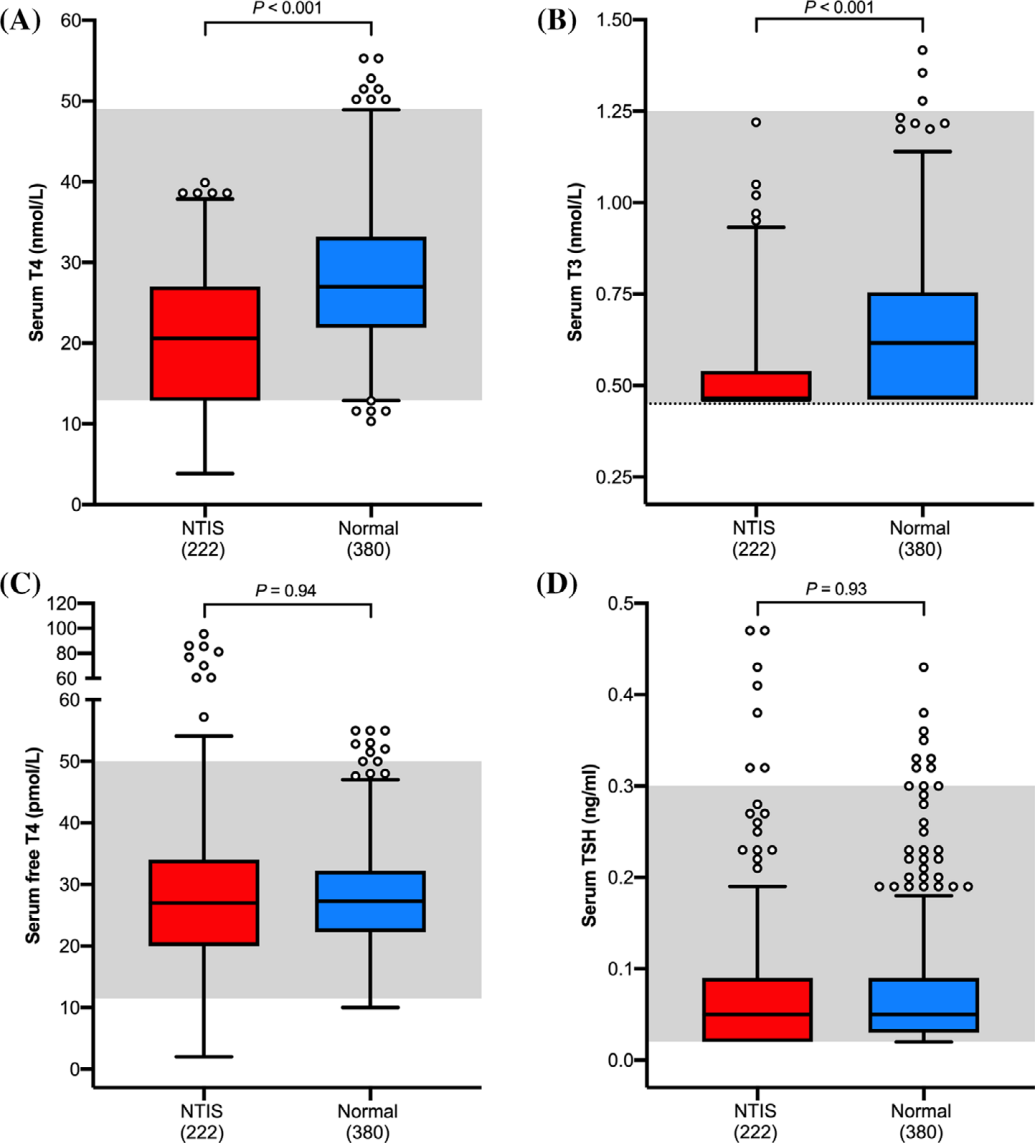
Boxplots of serum thyroid hormone concentrations in 222 cats with nonthyroidal illness and 380 clinically normal cats. A, Total thyroxine (T_4_); B, Triiodothyronine (T_3_); C, Free thyroxine (fT_4_) by dialysis; and, D, TSH. Boxes represent the interquartile range from the 25th to 75th percentile. The horizontal bar in each box represents the median value. The whiskers indicate the range of data values unless outliers are present, in which case the whiskers extend to a maximum of 1.5 times the interquartile range.^45^ Such outlying data points are represented by open circles. The shaded area indicates the reference interval. To convert serum T_4_ from nmol/L to μg/dL and fT_4_ from pmol/L to ng/dL, divide given concentrations by 12.87. To convert serum T_3_ from nmol/L to ng/dL, multiply given concentrations by 65.1. TSH, thyroid‐stimulating hormone

Cats with NTIS had lower serum T_4_ concentrations (median = 20.6 nmol/L) than did the clinically normal cats (median = 27.0 nmol/L; *P* < .001; Figure 1A). Fifty‐one (23%) of the cats with NTIS had low serum T_4_ concentrations, and 171 (77%) cats had serum T_4_ concentrations within the reference interval (Figure 1A).

#### 3.2.2 Serum T_3_ concentrations

Cats with NTIS had lower serum T_3_ concentrations (median = 0.46 nmol/L) than did the clinically normal cats (median = 0.62 nmol/L; *P* < .001; Figure 1B). Serum T_3_ concentrations were undetectable (<0.55 nmol/L) in 164 (73.9%) of the cats with NTIS and in 191 (50%) of the clinically normal cats (*P* < .001).

#### 3.2.3 Serum fT_4_ concentrations

Serum fT_4_ concentrations in the cats with NTIS (median = 27 pmol/L) did not differ from concentrations in the clinically normal cats (median = 27.3 nmol/L; *P* = .94; Figure 1C). Serum fT_4_ concentrations were low in 11 (4.9%) of the cats with NTIS, within the reference interval in 198 (89.2%), and high in 13 (5.9%). The cats with NTIS had a higher prevalence of high serum fT_4_ concentrations than did the clinically normal cats (*P* = .02), with 8 of the 13 cats having serum fT_4_ concentrations >60 pmol/L. The 13 cats with high serum fT_4_ concentrations suffered from hepatic disease (4 cats), neoplasia (3 cats), gastrointestinal disease (2 cats), cardiac disease (1 cat), infectious disease (1 cat), neurologic disease (1 cat), and renal disease (1 cat). None of these cats had any clinical evidence for hyperthyroidism (eg, no palpable thyroid nodule).

#### 3.2.4 Serum TSH concentrations

Serum TSH concentrations in the cats with NTIS did not differ from those in the clinically normal cats (median for both groups = 0.05 ng/mL; *P* = .93; Figure 1D). Serum TSH concentrations were slightly high in 7 (3.2%) of the cats with NTIS and in 8 (2.1%) of the clinically normal cats (*P =* .43). Serum TSH concentrations were undetectable (<0.03 ng/mL) in 69 (31.1%) of the cats with NTIS and in 97 (25.5%) of the clinically normal cats (*P =* .09).

### 3.3 Serum thyroid hormone and TSH concentrations in cats with mild, moderate, and severe illness

**FIGURE 2:**
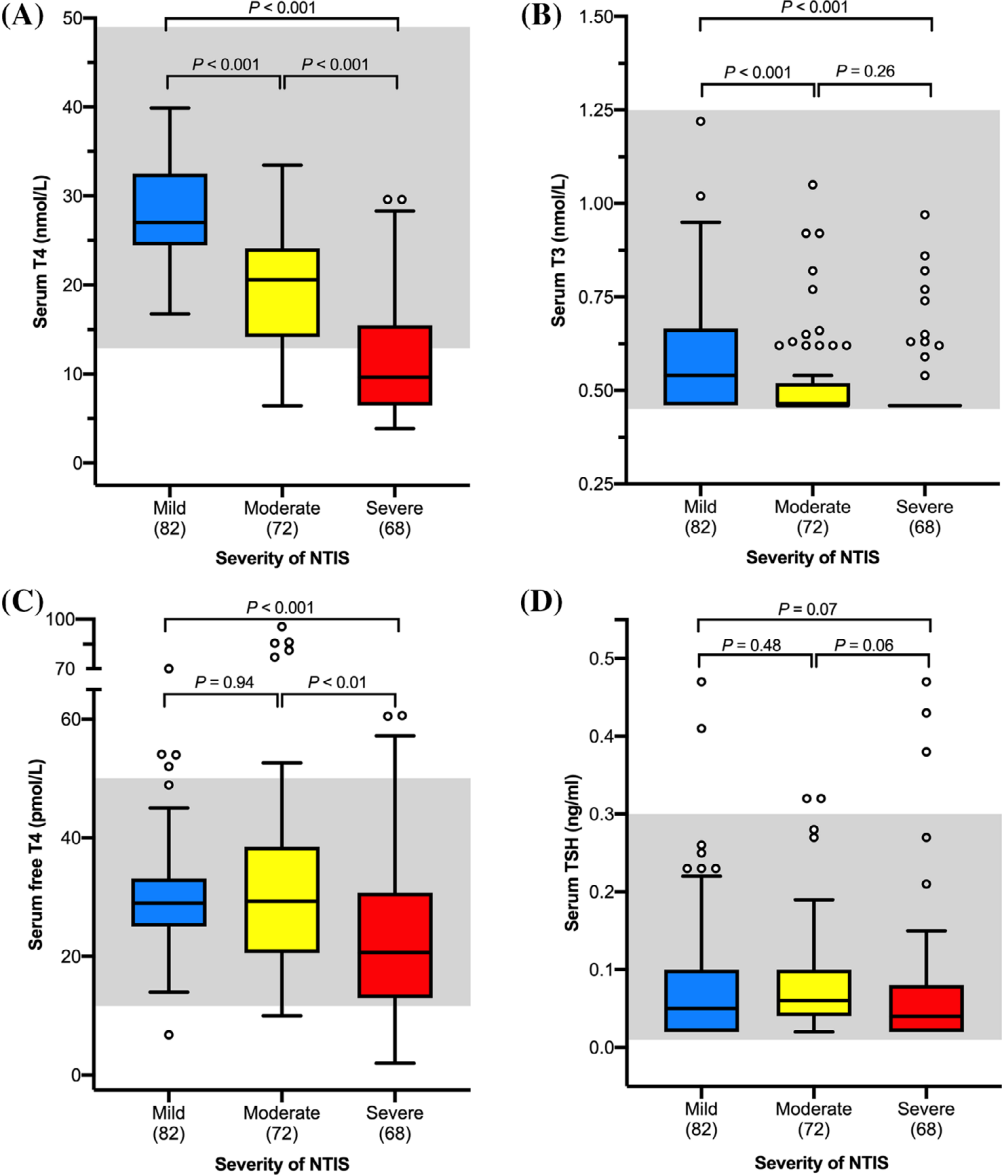
Boxplots of serum thyroid hormone concentrations in 222 cats with NTIS divided into groups according to severity of nonthyroidal illness. A, T_4_; B, T_3_; C, fT_4_ by dialysis; and, D, TSH. See Figure 1 for key. NTIS, nonthyroidal illness syndrome; TSH, thyroid‐stimulating hormone

When divided into groups based on severity of nonthyroidal illness (Figure 2), cats showed a progressive decrease in serum T_4_, T_3_, and fT_4_ concentrations (*P <* .001; Figure 2A‐C). All of the disease categories had cats with low T_4_, T_3_, and fT_4_ concentrations (Table 2).

#### 3.3.1 Serum T_4_ concentrations

The 68 cats with severe disease had lower serum total T_4_ concentrations (median = 9.7 nmol/L) than either the 72 cats with moderate disease (20.6 nmol/L, *P* < .001) or the 82 cats with mild disease (27 nmol/L; *P* < .001; Figure 2A). Cats with moderate disease had lower serum T_4_ concentrations than did the cats with mild disease (*P* < .001; Figure 2A). Nine of the 72 (12.5%) cats with moderate disease and 41 of the 68 (60.3%) cats with severe disease had low serum T_4_ concentrations (*P <* .001).

#### 3.3.2 Serum T_3_ concentrations

The cats with severe disease had lower serum T_3_ concentrations (median = 0.46 nmol/L) than did cats with mild disease (0.54 nmol/L; *P* < .001), and cats with moderate disease had lower serum T_3_ concentrations (0.46 nmol/L) than did those with mild disease (*P* < .001; Figure 2B). Serum T_3_ concentrations did not differ between cats with moderate and severe disease (*P* = .26; Figure 2B). Fifty of the 82 cats (61%) with mild disease, 57 of the 72 (79%) with moderate disease, and 57 of the 68 (84%) cats with severe disease had undetectable serum T_3_ concentrations (*P* = .03).

#### 3.3.3 Serum fT_4_ concentrations

Cats with severe disease had lower serum fT_4_ concentrations (median = 20.7 pmol/L) than did cats with either moderate (29 pmol/L; *P* < 0.01) or mild disease (29 pmol/L; *P* < .001). Serum fT_4_ concentrations did not differ between the cats with mild or moderate disease (*P* = .94; Figure 2C). None of the cats with mild disease, 1 of the 72 (1.4%) cats with moderate disease, and 10 of the 68 (14.7%) cats with severe disease had low serum fT_4_ concentrations (*P* < .001). In contrast, 4 of the 82 (4.9%) cats with mild disease, 6 of the 72 (8.3%) cats with moderate disease, and 3 of the 68 (4.4%) cats with severe disease had high serum fT_4_ concentrations (*P* = .55).

#### 3.3.4 Serum TSH concentrations

Serum TSH concentrations did not differ among the cats with mild (median = 0.05 ng/mL), moderate (0.06 ng/mL), and severe (0.04 ng/mL) nonthyroidal illness (*P* > .05, Figure 2D). However, when the mild and moderate groups were combined, serum TSH concentrations were higher in these 154 cats compared with the 68 cats with severe disease (*P* = .02).

Serum TSH concentrations were high in 2 (2.4%) of the 82 cats with mild disease, 2 (2.8%) of the 72 cats with moderate disease, and 3 (4.4%) of the 68 cats with severe illness (*P =* .77). Serum TSH concentrations were undetectable (<0.03 ng/mL) in 21 (25.6%) of the 82 cats with mild disease, 16 (22.2%) of the 72 cats with moderate disease, and 32 (47.1%) of the 68 cats with severe illness. A higher proportion of cats with severe disease had undetectable TSH concentrations compared with cats with mild and moderate disease (*P* < .001).

### 3.4 Serum thyroid hormone and TSH concentrations in cats with NTIS cats separated into 10 categories of disease

The cats with endocrine disease (all suffering from either severe diabetes or ketoacidotic diabetes mellitus) had lower serum concentrations of both T_4_ and fT_4_ (median = 10.9 nmol/L and 20 pmol/L, respectively) than did the cats in the other groups (Table 2; *P* < .01). However, compared to the other disease groups, the cats with endocrine disease also had the highest proportion (11/20; 55%; *P* < .01) of cats with severe illness (Table 2). Serum T_3_ and TSH concentrations did not differ among the cats with different categories of disease.

### 3.5 Serum thyroid hormone and TSH concentrations in cats alive or dead at 30 days

Of the 222 cats with NTIS, 56 (25.2%) cats died or were euthanized and 166 were alive at ≥30 days after thyroid testing. None of the 82 cats with mild illness died, compared with 12 (16.7%) of the 72 cats with moderate illness and 44 (64.7%) of the 68 cats with severe illness (*P* < .001).

**FIGURE 3:**
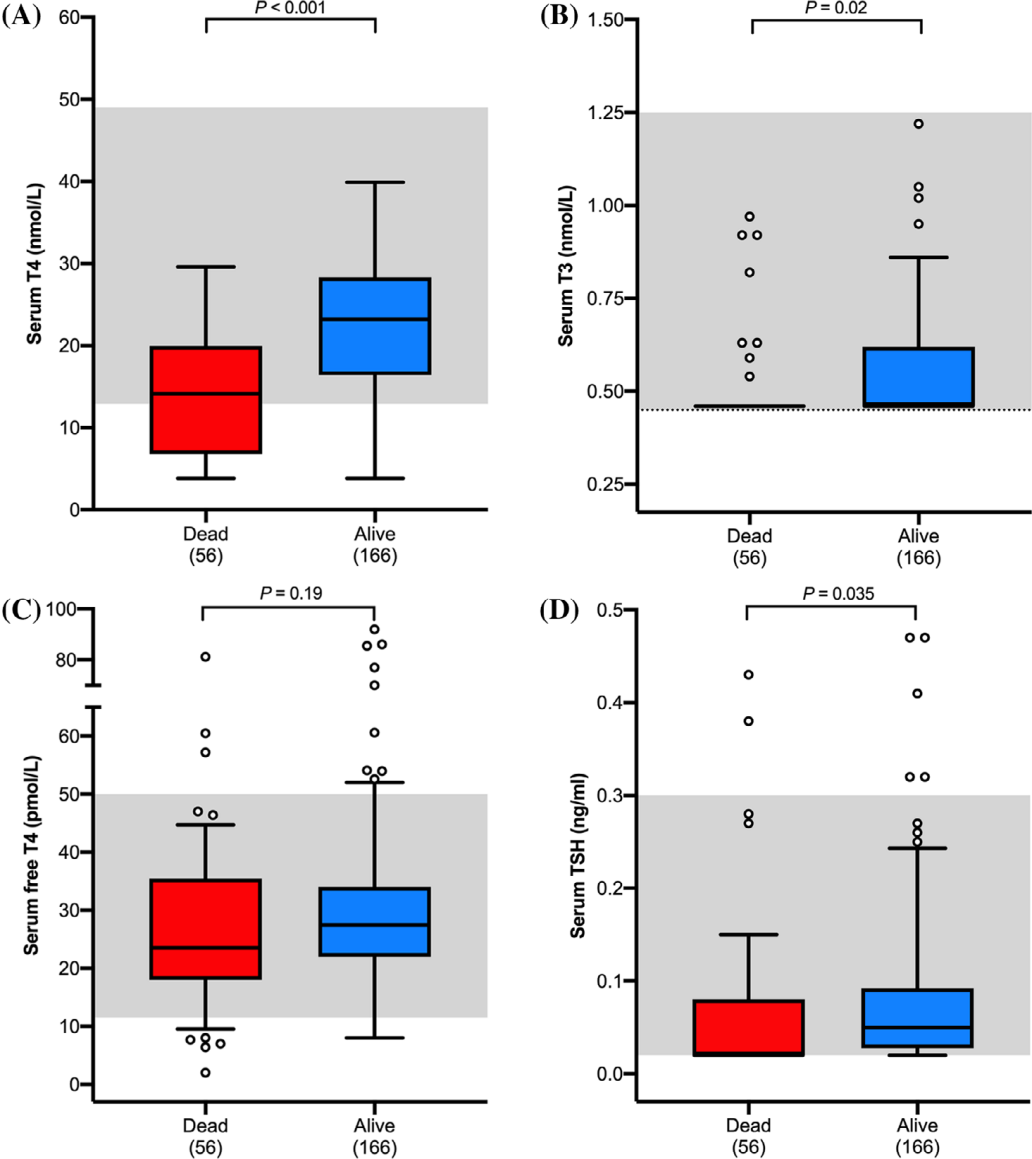
Boxplots of serum thyroid hormone concentrations in 222 cats with nonthyroidal illness divided into groups based on survival (dead vs alive) at 30 days after thyroid testing. A, T_4_; B, T_3_; C, fT_4_ by dialysis; and, D, TSH. See Figure 1 for key. TSH, thyroid‐stimulating hormone

The 56 cats that died or were euthanized had lower serum T_4_ concentrations (median = 14.2 nmol/L) than did the 166 cats that remained alive (23.2 nmol/L; Figure 3A; *P* < .001). Twenty‐four of the 56 (42.9%) dead cats had low serum T_4_ concentrations, compared with only 26 of the 166 (15.7%) cats that remained alive (*P* < .001).

Cats that died had lower serum total T_3_ concentrations (median = 0.46 nmol/L) than did cats that remained alive (0.46 nmol/L; Figure 3B; *P* = 0.02). Forty‐eight of the 56 (85.7%) dead cats had undetectable serum T_3_ concentrations, compared with 116 of the 166 (69.9%) cats that remained alive (*P* < .001).

Serum fT_4_ concentrations did not differ between the cats that died (median = 26.6 pmol/L) and those that remained alive (27.5 pmol/L; *P* = .19; Figure 3C). Three (5.4%) of the dead cats had high serum fT_4_ concentrations, compared with 10 (6%) of the cats that remained alive (*P* = .58). However, 7 (12.5%) of the cats that died had low serum fT_4_ concentrations, compared with 4 of the 166 (2.4%) cats that remained alive (*P* = .007).

Cats that died also had lower serum TSH concentrations (0.02 ng/mL) than did cats that remained alive (0.05 ng/mL; Figure 3D; *P* = .04). Twenty‐eight of the 56 (50%) dead cats had undetectable serum TSH concentrations, compared with only 41 of the 166 (24.7%) cats that remained alive (*P* < .001). Two of the 56 (3.6%) dead cats had high serum TSH concentrations, compared with 5 of the 166 (3%) cats that remained alive (*P* = .56).

### 3.6 Logistic regression model to predict 30‐day mortality outcome in cats with NTIS

A logistic regression analysis was performed to study the influence of several covariates (eg, age, sex, breed, T_4_, T_3_, fT_4_, and TSH) on 30‐day survival outcome. Age, sex, and breed were not significant (*P* > .3) and were excluded from the model. The final logistic regression model for 30‐day mortality in our cats included serum T_4_, T_3_, fT_4_, and TSH concentrations (Table 3).

**TABLE 3:** Logistic regression model predicting 30‐day mortality in 222 cats with nonthyroidal illness

Variable	Regression coefficient	Odd's ratio (95% CI)	*P* value	Area under ROC curve (95% CI)
Serum T_4_ (nmol/L)	−0.12	0.89 (0.85‐0.93)	<.001	0.789 (0.73‐0.84)
Serum T_3_ (undetectable vs within reference interval)	−0.45	0.63 (0.26‐1.56)	.32	—
Serum fT4 (pmol/L)	0.015	1.01 (0.99‐1.04)	.23	—
Serum TSH (undetectable vs within reference interval)	−0.90	0.41 (0.20‐0.82)	.01	—
Constant (intercept)	1.26	—	—	

Abbreviations: CI, confidence interval; fT_4_, free T_4_; ROC, receiver operating characteristic; T_3_, triiodothyronine; T_4_, thyroxine; TSH, thyroid‐stimulating hormone.

**FIGURE 4:**
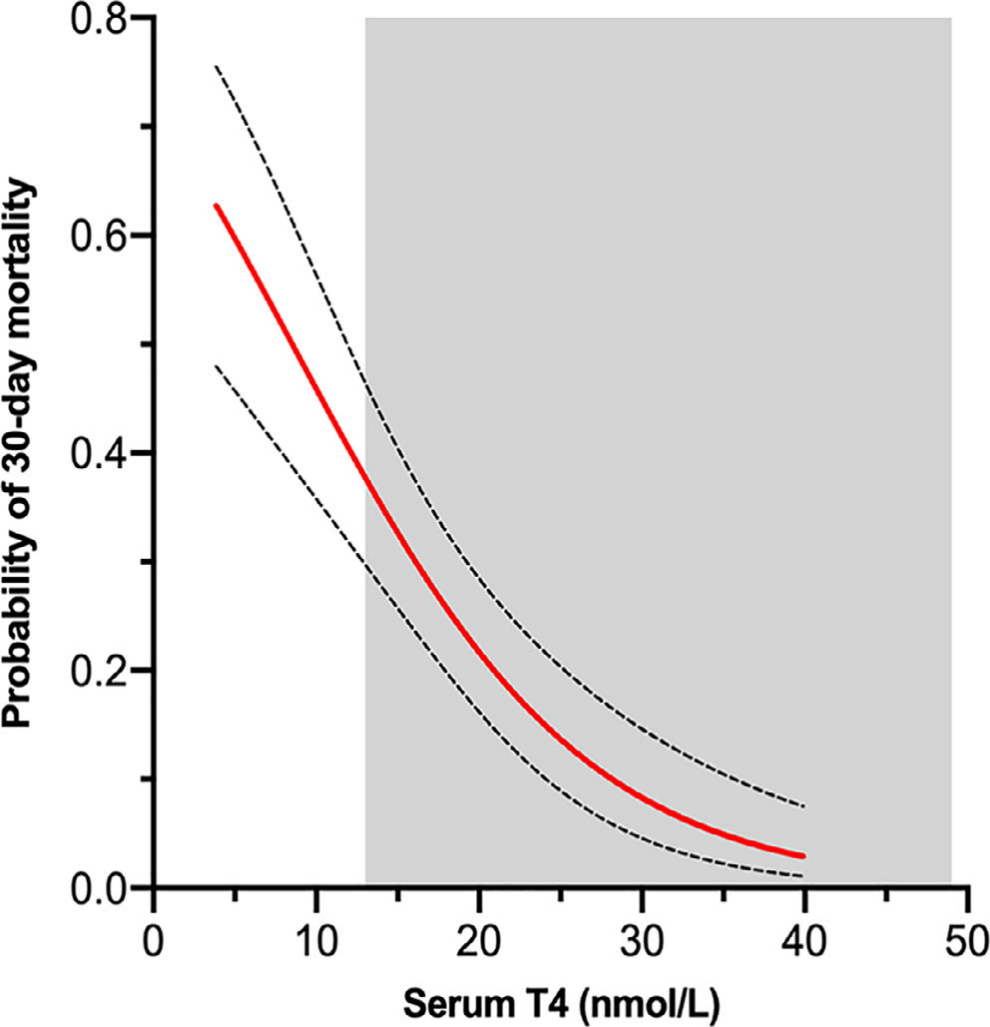
Probability of death within 30 days of testing as a function of serum T_4_ concentrations, according to our model of logistic regression analysis and 30‐day mortality (Log odds = 0.9623 − 0.1123*X). Dotted lines indicate the 95% confidence intervals. Shaded area represents the reference interval for serum T_4_ in cats

Of the 4 variables, serum T_4_ concentration showed the highest level of significance for predicting outcome (Table 3, Figure 4), with serum TSH concentration playing a lesser role in this model. The area under the ROC curve for this model was 0.789 (Table 3).

A classification matrix showing the distribution of our cats according to the observed and predicted outcome was derived from the model (Table 4). The test specificity for this model was high (87.4%), whereas the sensitivity of the model for predicting death was relatively low (46.4%).

**TABLE 4:** Comparison of actual survival outcome to outcome predicted by logistic regression analysis in 222 cats with nonthyroidal illness

		Predicted		Total	Percent correct
		Alive	Dead		
**Observed (actual)**	**Alive**	145	21	166	87.4^a^
	**Dead**	30	26	56	46.4^b^
**Total correctly classified (%)**					**77.0** ^c^

^a^ Test specificity.

^b^ Test sensitivity.

^c^ For this classification table, cutoff value of 0.4 was used for the predicted probability.

When serum T_4_ concentration was examined alone, the logistic regression equation (Log odds = 0.9533 − 0.1118*X) showed that for every 5 nmol/L (0.4 μg/dL) decrease in serum T_4_ concentration, there was a 56% increase in the odds of dying within the next 30 days (Figure 4).

## 4 DISCUSSION

Our results indicate that cats with nonthyroidal illness commonly develop low or undetectable serum concentrations of T_4_, T_3_, and TSH, the prevalence and extent of which increases with disease severity. As previously reported in cats with NTIS,^27^, ^28^, ^29^ our findings show that a variety of illnesses can suppress serum thyroid hormone concentrations, with the severity of illness having a much greater effect than the underlying disease itself. Our results of logistic regression modeling also indicate that lower serum T_4_ and undetectable TSH concentrations predict a lower likelihood of 30‐day survival in our cats with NTIS.

Serum T_4_ concentration has been the most common thyroid hormone test evaluated in past studies of cats with NTIS, all of which show lower serum concentrations compared to normal.^27^, ^28^, ^29^, ^30^ Serum T_3_ concentration has been evaluated in only a single study,^29^ which also reported lower concentrations in cats with severe illness, similar to our results. We could not, however, determine the true prevalence of low serum T_3_ concentration in our sick cats because the test sensitivity (lower detection limit) for the chemiluminescent T_3_ assay used in our study cannot differentiate low‐normal concentrations from truly low concentrations (ie, half of our normal cats had undetectable serum T_3_ concentrations, similar to the cats with NTIS). However, the prevalence of undetectable serum T_3_ concentrations was higher in our cats with NTIS compared to the clinically normal cats (74% vs 50%, respectively; *P* < .001), again suggesting that serum T_3_ concentration is decreased in cats with NTIS.

Serum fT_4_ concentration previously has been evaluated in only 2 studies of cats with NTIS,^28^, ^29^ and our results agree with the results of those studies. Cats with NTIS, like dogs and humans, usually maintain normal serum fT_4_ concentrations (measured by equilibrium dialysis) unless stricken with severe illness. On the other hand, a small proportion of euthyroid cats with NTIS develop high serum fT_4_ concentrations (6.3% and 12.2% in prior studies),^28^, ^29^ similar to the 5.9% prevalence in our study, and to observations in humans with NTIS.^8^, ^50^, ^51^ The cause of increased serum fT_4_ concentrations in NTIS is unclear. Because cats with NTIS are not clinically hyperthyroid, the high serum fT_4_ concentrations could represent an assay artifact, possibly caused by dialyzable compounds in NTIS sera that interfere with the assay.^51^, ^52^ Regardless, high serum fT_4_ concentrations represent a temporary response to illness, with fT4 decreasing to normal as NTIS resolves.^51^, ^52^

In human patients with NTIS, changes in serum TSH concentrations are dynamic over time and markedly influenced by the severity of the illness. In human patients with mild illness, normal serum concentrations TSH are maintained.^1^, ^2^, ^3^, ^4^, ^5^ As severity of illness worsens, serum TSH concentrations decrease below the reference interval. Such low TSH concentrations in patients with low serum T_4_ and T_3_ concentrations indicate altered thyroid hormone negative feedback at the pituitary or hypothalamus, consistent with a state of central hypothyroidism.^3^, ^4^, ^8^, ^13^ As human patients recover from severe nonthyroidal illness, serum TSH concentrations increase and may transiently increase above the reference interval, a situation that can make NTIS difficult to distinguish from primary hypothyroidism.^3^, ^4^, ^8^, ^14^, ^53^

Serum TSH concentrations have not been examined in cats with NTIS, except for 2 small studies of cats with mild‐to‐moderate chronic kidney disease, both of which reported that serum TSH concentrations remained within the reference interval.^35^, ^36^ Similarly, serum TSH concentrations remained within the reference interval in most cats with NTIS in our study, but a third had undetectable concentrations (<0.03 ng/mL), a situation that reflects the low serum TSH concentrations that develop in humans with severe NTIS.^1^, ^2^, ^3^, ^4^, ^8^, ^13^ We could not determine the prevalence of truly low serum TSH concentrations in our sick cats, however, because the test sensitivity (lower detection limit) for the TSH assay used in our study fails to differentiate low‐normal concentrations from truly low concentrations (ie, many of our clinically normal cats had undetectable serum TSH concentrations similar to the cats with NTIS). That said, the prevalence of undetectable serum TSH concentrations was higher in our cats with NTIS, compared to the clinically normal cats (31% vs 25.5%, respectively), suggesting that serum TSH concentration may be truly low in some cats with NTIS. As in humans,^1^, ^2^, ^3^, ^4^, ^8^, ^13^ cats with severe illness had a higher prevalence of undetectable serum TSH concentrations than did cats with mild or moderate NTIS (*P* < .001).

Approximately 3% of cats with NTIS had high serum TSH concentration, with no clear relationship between severity of illness and death or recovery. Furthermore, the prevalence of high serum TSH concentrations in our sick cats did not differ from that of our clinically normal cats (2.1%; *P =* .43), suggesting that these high serum TSH concentrations may represent outlying results.^54^ We did not measure serial serum TSH concentrations in our cats, and thus it is not known if serum TSH concentrations decrease in cats with severe illness and increase (sometimes to slightly high concentrations) during recovery, as occurs in human patients.^3^, ^4^, ^8^, ^14^ Future studies of cats with NTIS to investigate changes in serum TSH concentrations over time of illness and recovery are needed to address this question.

Although such high serum TSH concentrations can make it more difficult to distinguish between cats with NTIS and those with iatrogenic or naturally occurring hypothyroidism (especially when serum T_4_ concentration is also low),^31^, ^32^, ^33^, ^34^ most cats with NTIS have serum TSH concentrations that are only slightly high (all <0.50 ng/mL in our study), whereas most reported cats with iatrogenic or naturally occurring hypothyroidism have much higher serum TSH concentrations (>0.9 ng/mL, or 3 times the upper limit of the TSH reference interval).^33^, ^34^, ^36^ Obviously, differentiating the cause of the high serum TSH concentrations (ie, NTIS vs hypothyroidism) is much more important in cats treated for hyperthyroidism that develop iatrogenic hypothyroidism, which is a common complication,^34^, ^36^ than in cats with naturally occurring hypothyroidism, which is a rare disorder.^32^, ^33^, ^34^

Cats that died within 30 days of thyroid testing had lower serum T_4_, T_3_, and TSH concentrations than did the cats that survived, suggesting that these hormone test results could be used to predict short‐term outcomes, as previously suggested for T_4_ in cats with NTIS.^27^, ^28^, ^30^ We used logistic regression analysis to show that both serum T_4_ and TSH concentrations could help predict survival, with serum T_4_ concentration being the main predictor (Table 3). In fact, odds of death increase by 56% for every 5 nmoL/L (0.4 μg/dL) decrease in serum T_4_ concentration from a baseline concentration of 40 nmol/L (Figure 4).

Our study had several limitations. First, we did not definitively rule out concurrent hyperthyroidism in our cats with NTIS with thyroid biopsy or thyroid scintigraphy. Only a few cats that recovered from their NTIS underwent follow‐up serum thyroid hormone testing, because such long‐term follow‐up was not part of our study design. Although all of our cats with NTIS were considered euthyroid on the basis of history and physical examination (ie, none had clinical signs of hyperthyroidism or palpable thyroid nodules), the reported prevalence of palpable thyroid nodules in proven hyperthyroid cats ranges from 79%^55^ to 98%,^56^, ^57^ so it is possible that we missed thyroid nodules in a few cats. It is also possible that an occasional cat had ectopic thyroid tissue that would not be identified by palpation. Although 13 cats had high serum fT_4_ concentrations, which could indicate hyperthyroidism, none showed consistent clinical signs. None of the NTIS survivors became clinically hyperthyroid after resolution of their NTIS. Therefore, the probability of having a large cohort of occult hyperthyroid cats, with thyroid disease being masked by NTIS, is, in our opinion, small.

Several cats with NTIS had multiple comorbidities, which made it difficult to categorize these cats into 1 of the 10 disease groups. In these cases, we allocated the cats to a particular disease group based on most important or severe disease, as determined both by the clinician examining the cat and primary author (M.E. Peterson). However, ultimately, disease group did not appear to be as important as severity of illness in determining the proportion of cats with suppressed serum T_4_ or TSH concentration, and therefore, predicting survival outcome.

The third limitation of our study concerns the poor analytic sensitivity of the commercial TSH assay. The assay has a lower limit of detection which is high enough to include both normal and low concentrations. Therefore, we could not differentiate low‐normal concentrations from truly low concentrations (ie, many of our clinically normal cats had undetectable serum TSH concentrations, similar to the cats with NTIS). A more sensitive, feline‐specific TSH assay, which could differentiate truly low serum TSH from low‐normal TSH concentrations, would help determine the value of assessing TSH when prognosticating about survival outcome in cats with NTIS.

In conclusion, our results indicate that cats with NTIS commonly develop low serum T_4_, T_3_, and TSH concentrations, the prevalence and extent of which increase with disease severity. In addition, we found that lower serum T_4_ and undetectable TSH concentrations both were associated with mortality and can be used to help predict survival outcome in cats with NTIS.

## CONFLICT OF INTEREST DECLARATION

Authors declare no conflicts of interest.

## OFF‐LABEL ANTIMICROBIAL DECLARATION

Authors declare no off‐label use of antimicrobials.

## INSTITUTIONAL ANIMAL CARE AND USE COMMITTEE (IACUC) OR OTHER APPROVAL DECLARATION

Authors declare that ethics approval (IACUC) was obtained before the study commenced.

## HUMAN ETHICS APPROVAL DECLARATION

Authors declare human ethics approval was not needed for this study.
